# Exploring consensus on how to measure smoking cessation. A Delphi study

**DOI:** 10.1186/s12889-017-4902-7

**Published:** 2017-11-21

**Authors:** Kei Long Cheung, Dennis de Ruijter, Mickaël Hiligsmann, Iman Elfeddali, Ciska Hoving, Silvia M. A. A. Evers, Hein de Vries

**Affiliations:** 1 Health Services Research, CAPHRI Care and Public Health Research Institute, Maastricht University, P.O. Box 616, Maastricht, the Netherlands; 2 Health Promotion, CAPHRI Care and Public Health Research Institute, Maastricht, the Netherlands; 3 GGzBreburg, Tilburg, the Netherlands; 4 Tranzo Department, Tilburg University, Tilburg, the Netherlands; 5 Trimbos Institute, Netherlands Institute of Mental Health and Addiction, Utrecht, the Netherlands

**Keywords:** Smoking cessation, Delphi, Consensus, Outcome criteria, Measure, Tobacco control, Biochemical validation, Self-report, Abstinence

## Abstract

**Background:**

Different criteria regarding outcome measures in smoking research are used, which can lead to confusion about study results. Consensus in outcome criteria may enhance the comparability of future studies. This study aims (1) to provide an overview of tobacco researchers’ considered preferences regarding outcome criteria in randomized controlled smoking cessation trials, and (2) to identify the extent to which researchers can reach consensus on the importance of these outcome criteria.

**Methods:**

A three-round online Delphi study was conducted among smoking cessation experts. In the first round, the most important smoking cessation outcome measures were collected by means of open-ended questions, which were categorized around self-reported and biochemical validation measures. Experts (*n* = 17) were asked to name the outcome measures (as well as their assessment method and ideal follow-up period) that they thought were important when assessing smoking-related outcomes. In the second (*n* = 48) and third rounds (*n* = 37), a list of outcome measures—identified in the first round—was presented to experts. Asking them to rate the importance of each measure on a seven-point scale.

**Results:**

Experts reached consensus on several items. For self-reports, experts agreed that prolonged abstinence (6 or/and 12 months), point prevalence abstinence (7 days), continuous abstinence (6 months), and the number of cigarettes smoked (7 days) are important outcome measures. Experts reached consensus that biochemical validation methods should not always be used. The preferred biochemical validation methods were carbon monoxide (expired air) and cotinine (saliva). Preferred follow-ups included 6 and/or 12 months, with or without intermediate measurements.

**Conclusions:**

Findings suggest only partial compliance with the Russell standard and that more outcome measures may be important (including seven-day point-prevalence abstinence, number of cigarettes smoked, and cotinine when using biochemical validation). This study showed where there is and is not consensus, reflecting the need to develop a more comprehensive standard. For these purposes we provided suggestions for the Russell 2.0 standard.

## Implications

Despite attempts to develop a standard set of outcome criteria, such as the Russell standard, studies still use different outcome measures. Consensus on the usefulness of outcome recommendations can be an important prerequisite for the wide implementation of any standard. Findings suggest that respondents are only partially compliant with the Russell standard and that more outcome measures may be important (including seven-day point-prevalence abstinence, number of cigarettes smoked, and cotinine when using biochemical validation). This study provides insight into where there is and is not consensus on tobacco-related effect measures in randomized controlled trials, and reflects the need to develop a more comprehensive standard.

## Background

Worldwide, the leading preventable cause of illness (e.g. lung cancer, respiratory- and cardiovascular diseases) and pre-mature death remains tobacco smoking. Smoking accounts for more than six million deaths every year in the world (via smoking-related diseases) [1]. More effort needs to be directed towards reducing smoking prevalence, and evidence-based, comprehensive tobacco control measures should be implemented [2, 3]. Hence, a wide array of effective interventions for smoking cessation has been developed [4–6].

Most interventions rely on randomized controlled trials [RCTs] for proof of efficacy. In doing so, they utilize a variety of smoking cessation outcome measures to evaluate the efficacy of interventions. This may limit the comparability of study results. Not only are various smoking cessation outcome measures used, previous literature has reported arguments for, and empiric evaluations of, specific measures [7, 8]. It seems that measures can be broadly classified as self-report and biochemical validation measures [8]. Examples of self-report measures are point-prevalence abstinence—the percentage of former smokers who are not smoking for a specific period of time (e.g. 24 h or 7 days) at the point of assessment—and continuous abstinence (the percentage of former smokers who remained abstinent since the introduction of an intervention or event) [8]. Examples of biochemical validation measures are carbon monoxide, which can be measured in expired air and blood, and cotinine, which is the major proximate metabolite of nicotine and can be measured in various biological specimens (e.g. saliva and urine) [9].

Velicer, Prochaska, Rossi, and Snow [7] reviewed outcome measures for smoking cessation and evaluated several self-report measures and biochemical validation measures. In the literature, self-report measures have been empirically compared and discussed [8]; several types of biochemical verification methods have been discussed as well [9]. Using different outcome measures (self-report and biochemical validation) may vary the reported abstinence rates more than twofold (e.g. Hurt et al. [10]). Selection of outcome measures should of course reflect the chosen study goals, which may lead to differences in outcomes between studies. However when possible, common outcome measures should be utilized in order to increase the comparability of effective interventions. According to West, Hajek, Stead, and Stapleton [11], depending on the criteria adopted, the success rates of trials can differ dramatically. However studies with differing measures, such as using or not using biochemical validation, or studies with different follow-up durations, have been combined in overviews [12–14]. Some studies report similarities in smoking cessation outcomes, like point-prevalence abstinence vs. prolonged abstinence, producing similar relative effect sizes [15]. Other studies, however, show differences in results, with point-prevalence abstinence producing smaller effect sizes than prolonged abstinence [16, 17]. Given that smoking cessation studies use different outcome measures, which limits the comparability and interpretability of their findings, a standard set of criteria for outcome measures in tobacco smoking research is needed to enable researchers to uniformly express their results [11].

Attempts have been made to develop a standard set of criteria for outcome measures that would be utilized by all investigators. A workgroup examined outcome measures used in clinical trials using a literature search in 2003 [16], resulting in an overview of abstinence measures and recommendations. Later, West et al. [11] set out criteria and proposed the Russell standard. It combines a period of prolonged prevalence/continuous abstinence (six months or 12 months) after the quit date, during which a participant is allowed up to 5 cigarettes. This is combined with a biochemical test, using expired air carbon monoxide. However, one may argue that the use of carbon monoxide also has limitations and that self-report is highly accurate except for high risk groups and medical patients [7]. Despite the proposed Russell standard, studies still use different outcome measures, resulting in Cochrane reviews using different outcomes of abstinence, such as 7-day point-prevalence abstinence after six months, three-month prolonged abstinence, and 12-month continuous abstinence [14, 18]. This clearly illustrates the lack of consensus about an optimal strategy and the need for a study assessing various views on outcome criteria, identifying where there is consensus.

Moreover, economic evaluations identify which and to what extent cessation interventions are cost-effective. This information may be valuable for decision-makers to prioritize the reimbursement of interventions. However, the effectiveness of interventions used in these analyses is based on different outcomes of abstinence as well [5, 19]. Hence, despite the existence of the Russell standard, in order for a standard to be used, consensus on the usefulness of the recommendations is also a prerequisite, consensus that may only partly exist for the Russell standard. Researchers need more standardisation regarding the measurement of smoking cessation to enhance interpretations of effectiveness and studies of cost-effectiveness. Exploring where there is consensus and where there is lack of consensus is thus important to enhance uniformity by adjusting or creating a standard set of criteria.

To date, no study has investigated the preferences of smoking cessation researchers and the extent of consensus regarding outcome criteria in randomized controlled smoking cessation trials. This study explored to what extent smoking cessation experts agree on the most important outcome criteria in RCTs. Consensus in outcome criteria, and thus deciding to use these outcome criteria, may enhance the comparability of future studies. Hence, the aim of the study is (1) to provide an overview of researchers’ opinions regarding the preferred outcome criteria (i.e. outcome measure, duration of abstinence or assessment method, and ideal follow-up) to be considered in smoking cessation RCTs, and (2) to identify the extent to which researchers have consensus on the importance of these outcome criteria. The results will reveal to what extent expert opinions are in agreement with the current Russell standard, and may indicate other potentially important outcome measures that should be considered.

## Method

A three-round online Delphi study was conducted among smoking cessation researchers using Formdesk® and Qualtrics® between July 2015 and April 2016. Three iterations are often sufficient to collect the needed information and to elicit consensus [20, 21]. The Delphi technique is a widely used and accepted method for achieving convergence of expert opinions [22]. This technique is a method for consensus-building by using a series of questionnaires to collect data from experts [22–26]. In contrast to other data gathering and analysis techniques, the Delphi technique employs multiple iterations [27] in which the feedback process allows and encourages the selected experts to reassess their initial judgments about the information from previous iterations. Moreover, the Delphi technique is characterized by its ability to provide anonymity to respondents, a controlled feedback process, and the suitability of a variety of statistical analysis techniques to interpret the data [23, 26]. These characteristics reduce the effects of dominant individuals and certain downsides of group dynamics, such as manipulation or coercion to adopt or conform to a certain viewpoint [23, 26]. Furthermore, the Delphi methodology is practical, as experts from different parts of the world can be included due to its online character, and experts can complete each questionnaire at their own convenience, mitigating difficulties from non-matching schedules [26].

Smoking cessation researchers of RCTs were selected as experts for this study (described in the Delphi rounds). Experts were recruited via an e-mail, inviting the researchers to participate in an online Delphi study. Additionally, participants in the first round were asked to suggest relevant researchers for the second and third rounds. For non-responders, an e-mail reminder was sent after two weeks, followed by another reminder after approximately four weeks. During each round, the researchers were invited to respond to specific questions in an online survey. Each survey took about 10–20 min to complete, and rounds were iterative in nature. In this study, for each outcome criteria, abstinence of smoking is defined as having smoked no cigarettes at all during the specified period of time.

### First round

Smoking cessation researchers of RCTs were selected as experts for this study. In our systematic search for experts, we used the PubMed database for authors of relevant papers. We filtered for English language papers from the last 10 years and relied on the following keywords: ‘smoking cessation AND RCT’, ‘smoking cessation AND randomized controlled trial’ and ‘smoking cessation AND randomized controlled trial’. Titles were screened for relevance, and when author information was available, all authors of these studies were selected for recruitment. In addition, experts were identified via Google Scholar search and the international network of the authors. The Google Scholar search was a scoping search using a similar search strategy as the PubMed database search, to make sure key smoking cessation researchers were included. This led to a list of 250 experts (randomized using Microsoft Excel® (via the RAND() function)), from which we invited a random sample of experts to participate in all three rounds of the Delphi study. We initially invited 30 experts to participate. Two weeks later, we invited 14 more experts (thus inviting 44 in total) to reach a sufficient number of participants, as we wanted to include at least 15 experts in the first round. This resulted in 17 participating experts (38.6% response rate) from seven countries (i.e. United States (US), Hong Kong, United Kingdom (UK), Germany, Sweden, Australia, and the Netherlands) for the first-round questionnaire. This number was deemed sufficient, as 10 to 15 experts are regarded as sufficient if the experts’ backgrounds are rather homogeneous [28]. As described by Ludwig [29], 15 to 20 participants have been used in the majority of Delphi studies.

In the first round, we collected the most important smoking cessation measures by means of open-ended questions. The survey consisted of two parts. First, experts were asked to answer a few items regarding demographic characteristics: gender (male, female), age, and current profession (post-doc researcher, assistant professor, associate professor, professor, senior researcher, and other). Second, open-ended questions were categorized around two themes: self-reported outcome measures and biochemical validation methods. For self-reported outcome measures we asked the following question: “What are according to you the most important self-reported outcome measures to assess smoking cessation in randomized controlled smoking cessation trials?” In the survey, there was a limitation of six answers to stimulate reporting of the most important criteria. Experts were asked to name the outcome measure (e.g. prolonged abstinence), its duration of abstinence (e.g. six months), and the ideal follow-up period (e.g. 6 and 12 months). For biochemical validation methods, the following question was addressed: “What are according to you the most important biochemical validation methods to assess smoking cessation in randomized controlled smoking cessation trials?” Experts were asked to name the outcome measure of the validation method (e.g. cotinine) and its assessment method (e.g. saliva samples). Additionally, they were asked to indicate for each outcome measure (i.e. both self-report and biochemical validation measures) that they reported whether there is a specific research population for which this measure would be inappropriate. Finally, experts were asked whether they had other comments and suggestions for smoking cessation experts for the following rounds of the present Delphi study.

The collected responses resulted in a list of smoking cessation measures that were indicated to be most important in randomized smoking cessation trials. Two researchers analysed this list of measures and where possible, merged measures that were semantically similar. After discussion with one more researcher, all three researchers fully agreed about the measures that were included in the second-round questionnaire.

### Second round

All 250 identified experts, plus researchers suggested by first round participants, were invited to participate in the second round. Of the 256 invited experts, 48 from 16 countries (i.e. Australia, France, UK, Canada, China, Germany, Greece, India, Israel, Italy, Malaysia, New Zealand, the Netherlands, Turkey, UK, and the US) completed the questionnaire, resulting in a 19% response rate. Experts were presented with a list of smoking cessation measures that were identified as most important in randomized smoking cessation trials during the first round. Then experts were asked to rate the factors in order of importance using a 7-point Likert scale ranging from 1 (not at all important) to 7 (extremely important). Factors such as outcome measures, abstinence duration/assessment method, and ideal follow-up period were evaluated. The survey assessed the ratings for self-report outcome measures, biochemical outcome measures, and ideal follow-up separately. For self-report and biochemical outcome measures, first the outcome measures were rated, which was then followed by the ideal abstinence duration per measure. Experts rated all factors due to forced response in the survey. To analyse the importance of each factor, the median score (Mdn) was calculated and a score of ≥6 was considered important (i.e. agreement with the factor being important) [30]. To gain an indication of the degree of consensus between experts on the factors, interquartile ranges (IQR) were calculated [30]. Using a 7-point Likert scale, IQRs with a value of ≤1 (i.e. more than half of the opinions fall within one point of the scale) indicate good consensus among the experts [24].

### Third round

Factors with IQRs of ≤1 were removed from the questionnaire for the third round. All experts from the second round were invited to re-rate the remaining factors for which there was no consensus. Again, experts rated all factors due to forced response in the survey. Of 48 invited experts, 37 experts from 12 countries (i.e. Australia, UK, Canada, China, Germany, Greece, India, Israel, Malaysia, New Zealand, the Netherlands, and the US) completed the questionnaire in this final round (77% response rate). For each factor in the third round questionnaire, the Mdn and IQR of the second round were presented alongside the question to re-rank the remaining factors/outcome measures on their importance.

## Results

In the first round, 86 unique factors were identified by 17 experts, grouped by self-report outcome measures (24 factors), biochemical validation methods (16 factors), combinations of outcome measures (37 factors), and the ideal follow-up periods (nine factors). In the second round, experts reached consensus (IQRs ≤1) on eight factors that were considered important (Mdn ≥ 6) (i.e. five factors for self-report outcome measures, two factors for biochemical validation methods, and one for the ideal follow-up periods). In the third round, experts reached consensus on 12 additional factors that were considered important (i.e. four for self-report outcome measures, two for biochemical validation methods, and 4 for the ideal follow-up periods). In the third round, it was revealed that there was consensus among experts that biochemical validation should not always be used to validate self-report smoking cessation measures (Mdn = 5, IQR = 1). In total, from all factors, experts reached consensus on 77 factors (90%), of which 20 factors were considered important (23%). The unique factors and results of the second and third rounds are depicted in Tables 1, 2, and 3.

**Table 1:** Importance and consensus on self-reported outcome measures (R2: *n* = 48, R3: *n* = 37)

Factors	R2: Mdn	R2: IQR	R3: Mdn	R3: IQR
Number of cigarettes smoked (past 24 h)	5.00	2.00	5.00	1.00
Number of cigarettes smoked (past 7 days)^a^	6.00	2.00	6.00	1.00
Quit attempts during past 7 days	4.00	2.00	4.00	1.00
Quit attempts past month	5.00	2.00	5.00	1.00
Point-prevalence abstinence^a^	6.00	2.00	6.00	1.00
• Past 24 h	5.00	3.00	5.00	1.00
• Past 7 days^a^	6.00	2.00	6.00	1.00
• Past 30 days	5.00	2.00	5.00	1.00
• Past 2 months	4.00	2.00	4.00	1.00
• Past 3 months	4.00	2.00	4.00	1.00
Continuous abstinence^a^	6.00	1.00	–	–
• Past 30 days^a^	6.00	2.00	6.00	1.00
• Past 2 months	5.00	2.00	5.00	1.00
• Past 3 months	5.00	2.00	5.00	0.00
• Past 6 months^a^	6.50	1.00	–	–
• Past 12 months	6.00	2.00	6.00	2.00
Prolonged abstinence^a^	7.00	1.00	–	–
• Past 30 days	5.00	2.00	5.00	1.00
• Past 2 months	4.50	1.00	–	–
• Past 3 months	5.00	2.00	5.00	1.00
• Past 6 months^a^	7.00	1.00	–	–
• Past 12 months^a^	6.50	1.00	–	–
Grace period:				
• 1 week, duration at the start of the quit attempt (grace period)	5.00	2.00	5.00	1.00
• 2 weeks, duration at the start of the quit attempt (grace period)	5.00	2.00	5.00	2.00

*Mdn* median score, *IQR* interquartile range

^a^ = Consensus (IQRs ≤1) on important items (Mdn ≥ 6)

**Table 2:** Importance and consensus on biochemical validation methods (R2: n = 48, R3: n = 37)

Factors	R2: Mdn	R2: IQR	R3: Mdn	R3: IQR
Anabasine, using urine samples	4.00	2.00	4.00	2.00
Carbon monoxide^a^	6.00	3.00	6.00	1.00
The assessment method to detect carbon monoxide:				
• Expired air samples^a^	6.00	2.00	6.00	1.00
• Blood samples	4.00	3.00	4.00	1.00
Cotinine^a^	6.00	1.00	–	–
• Saliva samples^a^	6.00	1.00	–	–
• Urine samples	5.00	2.00	5.00	1.00
• Blood samples	4.00	2.00	4.00	0.00
• Hair samples	4.00	2.00	4.00	1.00
• Plasma samples	4.00	2.00	4.00	1.00
• Serum samples	4.00	1.00	–	–
Thiocyanate	4.00	2.00	4.00	1.00
The assessment method to detect thiocyanate:			4.00	1.00
• Plasma samples	4.00	1.00	–	–
• Saliva samples	4.00	1.00	–	–
• Urine samples	4.00	2.00	4.00	0.00

*Mdn* median score, IQR interquartile range

^**a**^ = Consensus (IQRs ≤1) on important items (Mdn ≥ 6)

**Table 3:** Importance and consensus on duration of follow-up periods

Factors	R2: Mdn	R2: IQR	R3: Mdn	R3: IQR
At the set quit date or the end of a grace period:	4.00	3.00	4.00	1.00
Follow-up for 30 days	5.00	2.00	5.00	1.00
Follow-up for 3 months	5.00	2.00	5.00	1.00
Follow-up for 6 months^a^	6.00	2.00	6.00	1.00
Follow-up for 12 months^a^	6.00	2.00	6.00	0.00
Follow-up for 3 months with intermediate measurements	4.50	1.00	–	–
Follow-up for 6 months with intermediate measurements^a^	6.00	1.00	–	–
Follow-up for 12 months with intermediate measurements^a^	6.00	2.00	6.00	1.00
Follow-ups for 6 and 12 months^a^	6.00	2.00	6.00	0.00

*Mdn* median score, *IQR* interquartile range

^a^Consensus (IQRs ≤1) on important items (Mdn ≥ 6)

### Self-reported outcome measures

After the third round, experts reached consensus on four important self-reported outcome measures (point-prevalence abstinence, number of cigarettes smoked, continuous abstinence, and prolonged abstinence) and five related abstinence periods (see Table 1).. The median score for prolonged abstinence was higher than the other outcome measures, indicating higher importance. We revealed for point-prevalence abstinence that the important duration of abstinence was past seven days. Moreover, the number of cigarettes for the past seven days was deemed important as well. For continuous abstinence, past 30 days and past six months were both considered important, although past six months had a higher median score. For prolonged abstinence, the past six months and past 12 months were important, although here the past six months had a higher median score as well. No consensus was reached for the most important grace period for prolonged abstinence.

### Biochemical validation methods

Experts reached consensus that biochemical validation methods should not always be used to validate self-report smoking cessation measures (Mdn = 5, IQR = 1). In total, experts reached consensus on four important biochemical validation methods (see Table 2), with carbon monoxide and cotinine being important validation methods. For carbon monoxide, expired air samples were the preferred assessment method. Saliva samples were the most preferred assessment method for cotinine. Experts reached consensus that thiocyanate is not an important biochemical validation method.

### Ideal duration of follow-up periods

Experts reached consensus on five important factors regarding the ideal duration of follow-up periods (see Table 3); six months, 12 months, six months with intermediate measurements, 12 months with intermediate measurements, and six and 12 months. It seems that there was a higher consensus among experts regarding a follow-up of 12 months and a follow-up of six and 12 months (IQR = 0), compared to the other follow-up periods.

## Discussion

### Findings regarding expert opinion

In order to enhance comparability of studies, this study attempted to provide an overview of researchers’ top preferences and the consensus among researchers regarding the outcome criteria to be considered in randomized controlled smoking cessation trials.

With regard to the most important self-report outcome measure, our results show that researchers reached consensus that point prevalence abstinence, continuous abstinence, and prolonged abstinence are all important; each method having its own strengths and limitations as reported in the literature [8]). However, prolonged abstinence seems to be the most preferred outcome measure, especially six-month prolonged abstinence. Consensus on the grace period was not reached. Moreover, a seven-day point-prevalence abstinence and a 30-day or six-month continuous abstinence outcome measure were also regarded as important. Regarding the follow-up, results reflect that our respondents regarded six month or 12 month follow-up as most important.

For the usage of a standard, consensus on the usefulness of the recommendations is a prerequisite, which may only partly exist for the Russell standard. This study therefore sheds light on where expert opinion may differ from this standard (see Table 4). First, findings indicate the importance of including six-month prolonged abstinence, which is consistent with the Russell standard [11]. However, in contrast with the Russell standard, results showed that our respondents also deemed seven-day point-prevalence abstinence, six-month continuous abstinence, and number of cigarettes smoked after seven days important to include in a trial. It is not clear from this study whether these measures—regarded as important—should be used complementarily or alternatively. However results seem in line with recommendations from Hughes and colleagues (2003 [16]; 2010 [15]) to report prolonged abstinence as the preferred measure and point-prevalence abstinence as a secondary measure. These two measures complement each other as prolonged abstinence is more stable and a better indicator for lifelong abstinence and health benefit, while point-prevalence abstinence may suffer less from memory bias and missing data, and also detects delayed quitting [7, 16]. Consistent with the Russell standard, the ideal grace period remained undefined [16].

**Table 4:** Recommendations compared to the Russell standard

	Russell standard		Findings		Recommendations (Russell 2.0)	
Self-report	Prolonged abstinence	Over the whole follow-up period	Prolonged abstinence	6 or/and 12 months	Prolonged abstinence	6 or/and 12 months
			Point prevalence abstinence	7 days^a^	Point prevalence abstinence	7 days^a^
			Continuous abstinence	6 months^a^		
			Number of cigarettes smoked	7 days^a^	Number of cigarettes smoked	7 days^a^
Grace period		Undefined		Undefined		Two weeks
Biochemical validation	Carbon monoxide	(9 p.p.m. in) expired air	Carbon monoxide	expired air		
			Cotinine^b^	saliva^a^	Cotinine^b^	(15 ng/ml in) saliva^a^
Follow-up		6 or/and 12 months		6 or/and 12 months		6 or/and 12 months

^a^Different from the Russell standard

^b^Use biochemical validation in new product and harm-reduction studies

Second, concerning the use of biochemical validation methods our respondents indicated that they are not always needed in smoking cessation RCTs. Hence, this may differ from the Russell standard (see Table 4), in which a biochemical validation with carbon monoxide is recommended [11]. It is yet unclear under which conditions these biochemical validation methods are regarded as important by the researchers to support self-report outcomes. The SRNT Subcommittee on Biochemical Verification (2002), provides some guidance and recommends using biochemical validations in most new product and all harm-reduction studies, with the exception of circumstances where its use is not desirable or feasible, such as online data gathering [9]. When including biochemical validation methods, findings suggest that carbon monoxide (using expired air samples) and cotinine (using saliva samples) are most important. This is only partly consistent with recommendations from the Russell standard. Findings thus indicate that cotinine was viewed as an important biochemical measure to validate smoking-related outcomes [31]. Both measures have their strengths and limitations, with cotinine having superior sensitivity and specificity, while carbon monoxide is easily assessed, detects smoking and not non-combustible forms of nicotine delivery (e.g. nicotine replacement therapy), and does not require storing body fluids samples [7, 11].These biochemical validation methods are limited in that they may only indicate point prevalence abstinence due to their short half-life [9]. Consistent with the literature, findings show that experts deem thiocyanate of lesser importance in trials because it has inadequate sensitivity and specificity [7]. A systematic review of the literature showed that self-reports may underestimate true smoking prevalence [31]. Hence, studies benefit from biochemical verification as it provides an objective alternative to reported estimates.

Last, according to the experts, the ideal duration of follow-up is six and/or 12 months, with or without intermediate measurements. Hence, follow-up should span a minimum of six months. This is consistent with the Russell standard (see Table 4), which recommends a follow-up of 6 or 12 months from the target quit date or the end of a predefined ‘grace period’ [11].Others also recommend tying all follow-ups to the quit date and reporting on 6- and/or 12-month abstinence rates [16].

### Recommendations (Russell 2.0)

This study showed that that there is consensus that some outcome measures may not be viable options for most smoking cessation RCT’s (e.g. 2-month point-prevalence abstinence), and that some measures may be important to consider (e.g. six-month prolonged abstinence and seven-day point-prevalence abstinence). Discussion is needed about whether more uniform measurement is possible by developing a new standard or adjusting the Russell standard, consistent with expert preferences. The authors of the Russell standard mentioned that in time, due to experience, the standard may need revisions [11]. This study yielded findings that suggest considering more outcome criteria in smoking cessation RCTs (compared to the Russell standard) (see Table 4).

As multiple considerations are mentioned, researchers may be bewildered by the array of options in measuring smoking cessation. These findings may then not enhance uniformity. Hence, based on the stated preferences of this Delphi panel and literature on outcome criteria, we provide several recommendations, supporting the proposition of an adapted Russell standard (Russell 2.0) (see Table 4). First, next to the prolonged abstinence (with a follow-up of 6 and/or 12 months), we suggest including 7-day point-prevalence abstinence, while assessing the number of cigarettes smoked in the past seven days for those who are not abstinent. The 7-day point-prevalence abstinence may be viewed as to some extent measuring a different construct than 6-month prolonged abstinence [8]. From that viewpoint, to assess smoking cessation one needs to include both prolonged abstinence and 7-day point-prevalence abstinence. These two measures complement each other. Prolonged abstinence is more stable and a better indicator of lifelong abstinence and health, while point-prevalence abstinence may suffer less from memory bias and missing data. Point-prevalence abstinence also detects delayed quitting, including relapsed smokers who decided to continue to quit smoking after relapse (thus reflecting the dynamic process of quitting, in contrast to continuous abstinence) [7, 16]. To define an ideal grace period (as this is unclear based on our findings), a recommendation of 2-weeks may be used [16]. When a prolonged abstinence measure is included, we suggest omitting continuous abstinence as only a small minority of smokers actually change in a linear manner—from smoking to non-smoking—without experiencing any lapses or relapses [8, 32]. Second, we recommend using biochemical validation at least in a sample of the population. We argue that carbon monoxide may not be the preferable biomarker as it may be highly vulnerable to environmental influences, especially in light smokers (who have relatively low levels of carbon monoxide related to tobacco use). Instead, we found that cotinine is a preferable biomarker due to its superior sensitivity and specificity (not influenced by diet and pollution exposure) [9]. When using cotinine, literature suggests that the preferred cut-off point used in cotinine is 15 ng/ml (85 nmol/L) in saliva [9]. When cotinine is used, it must be stressed that it is important to assess the presence of other forms of nicotine including nicotine replacement therapy and extensive exposure to second-hand smoke [9]. Hence, we recommend considering cotinine; if this is not possible, carbon monoxide would be the preferred second option [9, 31]. As mentioned, and in line with the Russell standard, we suggest that the ideal follow-up include at least a 6-month follow-up, with preferably an additional 12-month follow-up to show long-term (and thus more stable) abstinence.

### Limitations

We recognize that this Delphi study has its limitations. First, one limitation is that we could not take into account specific requirements that may have to be addressed by a specific RCT; it is obvious that such requirements also influence the selection process of the most important outcome parameters. This study did not aim to suggest a universal outcome that applies in all smoking cessation trials. Moreover, our findings clearly reveal a need to consider more outcome criteria than those suggested by the Russell standard. As with the Russell standard, it is expected that these outcome criteria are most applicable in trials with face-to-face assessments with smokers that include a designated quit date [11]. When there is lack of face-to-face contact, biochemical validation may be difficult or inappropriate to conduct. However, in some studies without face-to-face contact, biochemical validation was still conducted, for instance using test strips to measure cotinine in saliva [33]. The advantage of using clear standards is that the comparison of study outcomes will be enhanced. Our results, however, reveal a tension between this need and the needs of researchers to best characterize the effects of their treatments, as more outcome measures were suggested than identified in the Russell standard. Second, it is unclear to what extent these factors are ranked by relative importance and which measures should be included in RCTs next to each other. The same reasoning applies to factors where no consensus was reached. It is not clear to what extent these factors are less important to consider (e.g. a Mdn of 5 may indicate that a certain factor is important in some situations but not all). Perhaps for these factors, the importance depends on the situation, and consensus may only be reached if the conditions are specified. In healthcare research, studies are increasingly using conjoint analyses (e.g. discrete choice experiments and best-worst scaling surveys), which may provide opportunities to shed light on the relative importance of our findings [34, 35]. Third, another limitation is that this study may suffer from non-response bias, which may lead to limited generalizability. However, this qualitative study is explorative in nature, and the non-response rates were not exceptional as unsolicited questionnaires were used [36]. However this study cannot rule out selection bias of experts with a deviant opinion regarding the outcome criteria. The possibility remains that experts with a deviant opinion may feel more inclined to report on their views, a bias that most studies may encounter. Moreover, as we filtered for English articles in search of experts, this study is prone to selection bias for smoking cessation experts. Further research may be needed to assess the external validity of our findings, suggesting that the Russell standard may not be sufficient in many trials. Therefore, it is important to consider more outcome criteria. Fourth, selection bias of participants as experts may have occurred as selection was based on the authorship of relevant papers. However in the recruitment process we made explicit the goal of this study and our interest for input from smoking cessation experts. It is conceivable that most researchers receiving this e-mail did not participate because they did not feel they were smoking cessation experts. This may also explain the (rather high) dropout rate. Moreover, we checked the list of participants and concluded that important smoking cessation experts had participated. Last, besides the importance of using (a set of) comparable outcome measures, guidelines for addressing missing values are highly important as well. Complete case analysis has been replaced by penalized imputation (“missing = smoking”), but that may produce estimates that are too conservative, requiring more advanced strategies, such as multiple imputation [37].

## Conclusions

The findings suggest that regarding expert opinion, only partial compliance with the Russell standard is reported by experts, which is congruent with the reports of efficacy studies. Experts seem to deem more outcome criteria important for consideration in randomized controlled smoking cessation trials. Consequently, findings suggest the need to develop an adapted version, a Russell 2.0 standard, that includes more outcome measures, such as: (1) six-month prolonged abstinence (or continuous abstinence); (2) seven-day point-prevalence abstinence with the numbers of cigarettes smoked in these seven days; (3) biochemical validation, at least in a sample of the population, with a preference for cotinine assessments over carbon monoxide because of its greater sensitivity and specificity; (4) follow-ups after 6 months, and preferably also after 12 months.

## Abbreviations

IQR: Interquartile range
Mdn: Median
RCT: Randomized controlled trial
